# If You Believe, It May Come True: The Relationship and Mechanism Between Self-Occupation Stereotypes of Private Kindergarten Teachers and Their Turnover Intention in China-Mainland

**DOI:** 10.3389/fpsyg.2021.756099

**Published:** 2021-12-17

**Authors:** Feng Yang, Yang Han, Minyan Li

**Affiliations:** Teacher Education Department, Taishan University, Tai’an, China

**Keywords:** occupation stereotypes, private kindergarten teachers, turnover intention, personal control sense, professional identity

## Abstract

In China-Mainland, the turnover rate of private kindergarten teachers remains high for a long time. With 692 Chinese private kindergarten teachers as subjects, we applied a questionnaire survey to examine the relationship between self-occupation stereotypes held by private kindergarten teachers and their turnover intention and the underlying mechanisms. The structured equation model (SCM) was conducted to analyze data and revealed a significantly positive correlation between self-occupation stereotypes and turnover intention. Further analyses showed that on the individual level, personal control sense mediated the relationship between self-occupation stereotypes and turnover intention, and on the organization level, professional identity mediated the relationship between them. Additionally, self-occupation stereotypes were also related to turnover intention via the chain-mediating role of personal control sense and professional identity. The current research firstly clarified the acting paths between self-occupation stereotypes of private kindergarten teachers and turnover intention on both the individual and the organization levels. In practice, the research provided a novel perspective for policy makers to alleviate the turnover tendency of private kindergarten teachers.

## Introduction

Since the 1980s, Chinese government has begun to carry out the so-called “one-child policy” to prevent excessive population growth. According to the policy, a couple can only have one child in their lifetime. However, with the coming of the aging society in recent years, the original one-child policy was changed into the two-child policy that all married couples could have two children. As expected, the two-child policy caused a sudden rise of the number of kindergarten-age children and challenged the affordance of public private kindergartens in China. To improve the affordance of preschool education system, Chinese government has taken a series of measures to promote the development of private kindergartens (Li et al., 2011; Pan et al., 2018). Benefiting from huge market demands for kindergartens and supportive policies from government, the number of private kindergartens has increased rapidly in recent years (Lin, 2019). However, while the number of private kindergartens is growing rapidly, the turnover rate of private kindergarten teachers also remains quite high. For example, according to the news from Preschool Education Website, in 2020, the turnover rate of private teachers working in less than 1 year was high 41% (Preschool Education Website, 2020).

To alleviate the high turnover rate of teachers, it is necessary to make sense of the contributing factors behind the phenomenon. Previous research has explored the contributing effects of external circumstance factors, such as salary, professional development, social support and so on (Li et al., 2016; Pan et al., 2018). Recently, several research suggests that internal cognitive factors of private kindergarten teachers also play an important role in the turnover of teachers (Tang et al., 2015; Ye et al., 2018; Tian and Xu, 2020). More recently, Yang et al. (unpublished)^1^ initially examined the relationship between self-occupation stereotypes of private kindergarten teachers and the turnover intention. The results showed a significantly positive relationship between self-occupation stereotypes and turnover intention. The more private kindergarten teachers held negative self-occupation stereotypes, the more likely they left the kindergarten in the future. With regard to the underlying mechanism, previous research preliminarily revealed that on the individual level, personal control sense partially mediated the relationship between self-occupation stereotypes of private kindergarten teachers and turnover intention. However, prior literature found that negative attitudes toward jobs was significantly related to lower professional identity and lower professional identity may further induce employees’ turnover tendency (Mcardle et al., 2007; Li, 2014; Fisherman, 2015), thus indicating the possible mediating role of professional identity on the organization level between self-occupation stereotypes and turnover intention. That is to say, what accounts for the relationship between self-occupation stereotypes of private kindergarten teachers and turnover intention actually still is an open question. Given that, the current research would conduct a more comprehensive investigation to uncover the mechanism underlying the relationship between self-occupation stereotypes and turnover intention. Specifically, with 692 Chinese private kindergarten teachers as subjects, we applied a questionnaire survey to examine the relationship between self-occupation stereotypes and turnover intention, and the possible mediating roles of personal control sense and professional identity.

## Literature Review

According to the “cognitive miser” hypothesis (Fiske and Taylor, 1984), to make sense of our surrounding social world without being cognitively overwhelmed, we commonly simplify our information processing by applying stereotypes. In general stereotypes are defined as some socially shared beliefs that members of a social category commonly possess some typical traits or characteristics (Hilton and von Hippel, 1996). To date, researchers have revealed various of stereotypes, including but not limited to race, gender, age, and occupation stereotypes (Banaji and Hardin, 1996; Chasteen et al., 2002; Alt et al., 2019; Bhaskaran and Bhallamudi, 2019). An abundant literature suggests that upon activated, stereotypes can produce a wide downstream effect on individuals’ cognition and behaviors (for a review, see Fiske, 1998). For instance, in a classic study, researchers found that participants whose elderly stereotypes were primed walked more slowly down the hallway when leaving the lab than those without elderly stereotypes primed (Bargh et al., 1996). Similarly, the literature concerning gender stereotypes showed that females holding gender stereotypes were more likely to engage in stereotype-consistent jobs (i.e., secretary) than stereotype-inconsistent jobs (i.e., truck driver; Ellemers, 2018; Bhaskaran and Bhallamudi, 2019). In line with such previous stereotype literature, recent work by Yang et al. (unpublished, see text footnote 1) suggested that negative self-occupation stereotypes held by private kindergarten teachers could be harmful to their career development and there was a significant correlation between occupation stereotypes and their turnover intention. However, as we have mention above, the mechanism underling the phenomenon still remains unclear. In the current research, we proposed that personal control sense and professional identity may both mediated the relationship between occupation stereotypes and turnover intention. In the following section, we will review several key variables involved in the research and illustrate the relationships among them.

### Occupation Stereotypes

According to the definition by He et al. (2019), occupation stereotypes represent some preconceived beliefs that some characteristics should be commonly hold by people in an occupation, or an attitude tendency about a particular occupation or individuals who are employed in that occupation (He et al., 2019). With regard to the occupation stereotypes of private kindergarten teachers, they commonly refer to some preexisting beliefs that compared to being a teacher in a public kindergarten, being a teacher in a private kindergarten will be a more steady and promising job (Lin, 2019). In China, being a public school teacher means that he/she gets salaries from government finance—the so-called working “inside the system,” while being a private school teacher means that he/she will be paid by non-governmental organizations or individuals—the so-called working “outside the system.” Due to the influences of both historical and realistic factors (this issue will be further discussed in the “Discussion” section), across almost all industries (including the education industry) in China-Mainland, having a job paid by government is considered to be steadier and promising (Tan, 2009; Lin, 2019). To date, although there is less direct evidence about the relationship between self-occupation stereotypes and turnover intention, there actually are several studies in other industries suggesting the significant correlation between negative stereotypes and turnover intention (von Hippel et al., 2013; Park et al., 2014; Kim and Moon, 2021). For example, Park et al. (2014) found that, in South Korea, those male nurses who possess the stereotypes that females than males are more suitable for nursing displayed a significant turnover intention. The extensive existence of the downstream effect of stereotypes indicates it is necessary and reasonable for us to explore whether there will be a significant correlation between self-occupation stereotypes of private kindergarten teachers and their turnover intention.

### Personal Control Sense

Human beings have an innate desire for control over their environment (Langer, 1975). Control refers to the ability to influence outcomes in one’s environment (Skinner, 1996). There is extensive theoretical and empirical research suggesting that having control over surrounding circumstances is a basic need for humans’ healthy development and psychological well-being (Fiske and Dépret, 1996; Deci and Ryan, 2012; Landau et al., 2015). Personal control sense is so important that when people perceive a loss of control over their environment, they try their best to reinstate the control sense by various of methods, such as paying more attention to external circumstances (Kraus et al., 2009), seeking illusory correlations among a series of irrelevant events (Whitson and Galinsky, 2008), and even buying utilitarian products (Chen et al., 2017). Previous research suggested that if a private kindergarten teacher held negative occupation stereotypes that being a private kindergarten teacher is less steady and promising than being a public kindergarten teacher, he/she would perceive lower control sense over surrounding environments (Yang et al., unpublished, see text footnote 1). In this situation, some teachers may escape from the current environment so that they can reestablish their control sense in another workplace. That is, the self-occupation stereotypes of private kindergarten teachers induced their lower personal control sense, and this may further contribute to their turnover.

### Professional Identity

As a self-concept, professional identity refers to individuals’ awareness about the social impact and importance of their profession, it is the psychological basis for people to do their job well and achieve the organizational goal (Moore and Hofman, 1988; Zhang et al., 2018). Hong (2010) has documented that professional identity is an important factor in understanding individuals’ career and career decisions. People with a strong sense of professional identity tend to take pride in their career and achieve self-realization and growth through it (Butler and Constantine, 2005; Yu and Nilan, 2008). Past literature has demonstrated that the formation of professional identity depends on a range of factors, such as social context, cultural factors, career goals, job stress, salaries and so on (Sun and Qian, 2007; Rodgers and Scott, 2008). And lower professional identity has been found to be an important antecedent for the turnover of employees (Mcardle et al., 2007; Li, 2014). With 115 private kindergarten teachers as participants, Li (2014) employed a questionnaire survey and found that there was a significantly negative correlation between professional identity of private kindergarten teachers and their turnover intention. More importantly, in China, compared to public kindergarten teachers, private kindergarten teachers display a relatively lower professional identity level (Tan, 2009). So, it is necessary for researchers to pay more attention to the professional identity of private kindergarten teachers, and systematically explore the relationships among self-occupation stereotypes of private kindergarten teachers, professional identity, and turnover intention.

### Turnover Intention of Private Kindergarten Teachers in China-Mainland

Turnover intention refers to the probability that an employee voluntarily leaves his or her job in the period ahead (March and Simon, 1958). Past research has demonstrated that turnover intention is the principal cognitive precursor of turnover behavior with great explanatory power (Michaels and Spector, 1982; Zhang et al., 2018). For a teacher, having turnover intention does not mean that he/she will leave the current organization, but implies a thought of dosing so. A large number of past literature has analyzed the influencing factors of turnover intention of private kindergarten teachers, mainly including external environment factors and internal cognitive factors of teachers (Li et al., 2011; Tang et al., 2015; Pan et al., 2018; Ye et al., 2018; Tian and Xu, 2020). The former includes salary level, work stress, and welfare treatment, while the latter includes job satisfaction, professional identity, occupational commitment, and so on. Thus, to alleviate the high turnover rate of private kindergarten teachers in China-Mainland, besides the financial and political supports form Chinese government, it is also important to explore internal cognitive factors which contributes to the turnover of teachers (Yang et al., 2018).

### The Relationship Among Self-Occupation Stereotypes of Private Kindergarten Teachers, Personal Control Sense, Professional Identity, and Turnover Intention

According to the definition of the occupation stereotypes of private kindergarten teachers mentioned above, a central aspect of self-occupation stereotypes about private kindergarten teachers is that being a private kindergarten teacher is a less steady job than being a public kindergarten teacher. In this case, intuitively, self-occupation stereotypes held by private kindergarten teachers may induce their relatively lower personal control sense. Supporting our speculation, with a sample of 1991 undergraduate students, Duffy (2010) found that the preexisted negative attitudes toward their job was found to be associated with lower personal control sense, and lower control sense was further found to be significantly related to turnover intention, thus indicating the possible indirect role of personal control sense (Duffy, 2010; Ahn, 2015; Kilo and Hassmén, 2016). More recently, as mentioned in the beginning, Yang et al. (unpublished, see text footnote 1) recently have found that there was a significantly positive relationship between self-occupation stereotypes and turnover intention of private kindergarten teachers, and personal control sense mediated the relationship between them. However, as we have pointed out in the “Introduction” section, the research only revealed the mediating role of personal control sense on the individual level, leaving an open question what accounts for the relationship between them on the organization level. To fill this gap, the current research will conduct a more comprehensive investigation about the relationship between self-occupation stereotypes and the turnover intention of private kindergarten teachers, and attempt to reveal the underlying mechanism on both the individual and the organization levels.

On the basis of previous literature review, we proposed that besides personal control sense, professional identity also mediated the relationship between occupation stereotypes and turnover intention. For the relationship between self-occupation stereotypes and turnover intention, despite lacking direct empirical evidence, there are still some supportive results among past literature. As an example, Sun and Qian (2007) have documented that for private kindergarten teachers in China, the preexisting bias or negative attitude tendency toward their own organization and working environment would have a negative influence on their professional identity. Based on this, we speculated that negative self-occupation stereotypes held by private kindergarten teachers may reduce their professional identity toward the job. And lower professional identity has been found to be one possible reason causing the turnover of private kindergarten teachers in China (Li, 2014). Therefore, advancing previous research by Yang et al. (unpublished, see text footnote 1), the current research proposed that besides the mediating role of personal control sense, professional identity may also mediate the relationship between self-occupation stereotypes and the turnover intention of private kindergarten teachers.

**FIGURE 1:**
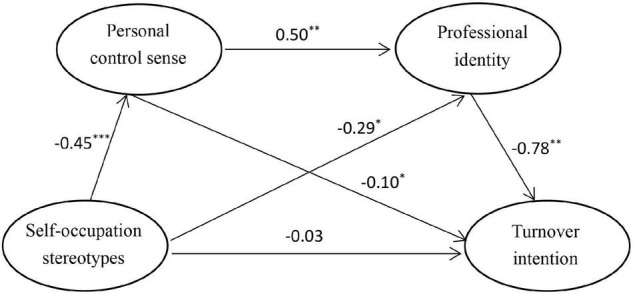
Multiple mediating roles of personal control sense and professional identity in the relationship between self-occupation stereotypes of private kindergarten teachers and turnover intention. Standard path coefficients were presented. *p < 0.05, ^**^p < 0.01, ^***^p < 0.001. *N* = 688.

In addition to the independent mediating roles of personal control sense and professional identity, with the purpose of exploration, we also attempted to examine the chain-mediating role of the above mediators between self-occupation stereotypes and turnover intention. Regarding the relationship between personal control sense and professional identity, recent research concerning kindergarten teachers’ role stress suggested that professional identity was more likely to decline when teachers perceived the role stress (Zhou et al., 2020). Concretely, role stress of a teacher often refers to a situation in which that a teacher can’t meet the demands or expectations from multiple sources (Yang et al., 2018), a state highly similar to low control sense due to failing to complete multiple tasks. So, it may be possible that the chain-mediation of personal control sense and professional identity exists between self-occupation stereotypes and turnover intention. Given that, we would make an exploratory investigation on the chain-mediating role of personal control sense and professional identity. The relationships among occupation stereotypes, personal control sense, professional identity, and turnover intention can be found in Figure 1.

## The Current Research

As we have documented, prior research has revealed that personal control sense partially mediated the relationship between occupation stereotypes and turnover intention on the individual level (Yang et al., unpublished, see text footnote 1), but it is still an open question about what accounts for the relationship between self-occupation stereotypes and the turnover intention of private kindergarten teachers on the organization level. To fill this gap, the current research would conduct a more comprehensive investigation on the relationship and the underlying mechanism between self-occupation stereotypes and turnover intention. Specifically, the following four hypotheses would be tested:

Hypothesis 1: There would be a significantly positive relationship between the self-occupation stereotypes of private kindergarten teachers and their turnover intention.

Hypothesis 2: On the individual level, personal control sense would mediate the relationship between self-occupation stereotypes and turnover intention.

Hypothesis 3: On the organization level, professional identity would mediate the relationship between self-occupation stereotypes and turnover intention.

Hypothesis 4: Both personal control sense and professional identity would act as the chain-mediating role between self-occupation stereotypes and turnover intention.

## Materials and Measures

### Participants and Procedure

In 2020, to improve the teaching level of private kindergarten teachers, an online training toward 720 private kindergarten teachers from multiple cities of Shandong Province was organized by Shandong Provincial Department of Education. During the training, through a convenient sampling, we collected the data of the research by an online questionnaire survey. Specifically, for the convenience of communication with each other, three WeChat group chats including all 720 teachers in total were established. We, respectively, initiated our questionnaire survey among three group chats. Before the formal survey, we introduced the academic purpose and non-commercial nature of this survey, and expressed our sincere welcome for their participation. On the basis of voluntary, 692 of 720 teachers finally took part in this survey.

We presented all items and collected data via a professional data-collection software—Sojump. Prior to the formal answer, we firstly introduced some attention points for participants when answering the items. If they did not have any question about the survey, they could complete the questionnaire on a computer or a telephone. It should be explained that according to the setting of the software, participants could not submit the questionnaire successfully until they completed all items. For this reason, no missing data generated for our database. However, because 4 participants indicated the same answer for all items, they were regarded as unqualified participants and excluded from the following data analyses. As a result, 688 participants (687 females; *M*_age_ = 28 years, *SD* = 7.5 years, range = 18–52 years) were included in final data analyses. In addition to the key variables involved in the research, some demographic information also was collected, such as salary, education level, gender, and working years. However, considering only 1 male participant involved in the survey, this variable was deliberately ignored in further data analyses. After all participants submitted their questionnaires successfully, we again expressed our gratitude and appreciation for their participation.

### Measures

#### Occupation Stereotypes About Private Kindergarten Teachers

Considering that there has been no scale to exclusively measure occupational stereotypes about private kindergarten teachers in China-Mainland, on the basis of relevant literature (Li, 2014; Pan et al., 2018; Lin, 2019), we developed a scale containing four items to measure occupation stereotypes about private kindergarten teachers. The items of the scale were listed as following: (1) compared to public kindergarten teachers, private kindergarten teachers generally have fewer opportunities to advance themselves; (2) being a private kindergarten teacher cannot satisfy my need for safety and stability; (3) compared to public kindergarten teachers, private kindergarten teachers acquire less respect from people around them; (4) at present, it is a common phenomenon for a private kindergarten teacher to regard the present job as a stepping-stone to a public kindergarten. For the self-designed scale, an exploratory factor analysis (EFA) was used to assess its reliability and validity. The EFA adopted orthogonal varimax rotation following a principal component analysis procedure. In line with previous research (Wang et al., 2020), several criteria were used to determine the number of factors, including Cattell’s Scree Test, eigenvalues greater than 1, and factor loading greater than 0.5. As a result, 1 factor was successfully extracted from 4 items and the factor loading of each item was greater than 0.5 (ranging from 0.74 to 0.80). Totally, 61.72% of variance was explained. In the current research, the internal consistency coefficient of the scale was 0.80, indicating a good internal consistency reliability (Kline, 1998).

#### Personal Control Sense

We assessed participants’ personal control sense over external circumstance using the control sense scale developed by Lachman and Weaver (1998). The scale includes 12 items and participants need to indicate their agreement for each item on the 7-point scale (e.g., I can do just about anything that I really set my mind; 1 = s*trongly disagree*, 7 = *strongly agree*), which has been successfully used in Chinese culture (Yang et al., 2016). The scale can be classified into two dimensions: personal mastery and perceived constraints. Following previous research (Kraus et al., 2009; Yang et al., 2016), we created a composite score to represent participants’ general control sense after reversely scoring those items assessing the perceived constraints. As a consequence, higher score indicated stronger control sense. The analysis of the internal consistency reliability showed a good reliability (α = 0.84).

#### Professional Identity

We measured participants’ professional identity via the professional identity scale of kindergarten teachers developed by Wang (2009). With kindergarten teachers as participants, previous research has demonstrated that the well suitability of the scale in Chinese culture (Wang et al., 2014). The scale contains 14 items and such items are classified into four dimensions (professional cognition, professional emotion, professional desire, and professional volition) to detect the professional identity of teachers (e.g., as a kindergarten teacher, I can acquire a great sense of achievement). Participants needed to indicate the extent to which they agreed with each item on a 5-point scale (1 = s*trongly disagree*, 5 = *strongly agree*), and higher scores represented higher professional identity. In the current research, the Cronbach’s α values for the four subscales were 0.76, 0.80, 0.71, and 0.80, which were acceptable according to the criterion by Kline (1998). The results of confirmatory factor analysis (EFA) showed an acceptable constructional validity of the four-dimension scale: χ*^2^*/*df* = 3.06, *CFI* = 0.96, *TLI* = 0.95, *RMSEA* = 0.05, *SRMR* = 0.05.

#### Turnover Intention

The turnover intention of private kindergarten teachers was detected through the scale developed by Farh et al. (1998). The scale consists of four items: (1) the idea of leaving the present job often comes to my mind; (2) I may leave the present organization and hunt for a new job in next year; (3) I intend to work permanently in the present organization (reversely scoring); and (4) if I continue to stay in the present organization, my work prospect may be not optimistic. Participants needed to indicate the extent to which they agreed with each description on a 5-point scale (1 = s*trongly disagree*, 5 = *strongly agree*). With kindergarten teachers as participants, prior literature has demonstrated the well suitability of the scale in Chinese culture (Ye et al., 2018). The coefficient of the internal consistency of the scale was 0.87, indicating a good reliability of the scale.

## Results

### Descriptive Statistics

Considering similar variables and research topic, we conducted data analyses and established the structural equation model (SEM) keeping in line with prior research by Zhou et al. (2020). Firstly, SPSS 23.0 was used to sort the database and provide preliminary data analyses. Then, AMOS 23.0 was used to perform the SEM to test the mediation paths between occupation stereotypes and turnover intention. Table 1 presents the means, standard deviations, and Pearson correlations among all variables (including three demographic variables) involved in the research. Supporting our hypothesis 1, the table showed that there was a significantly positive between self-occupation stereotypes and turnover intention. Additionally, except professional cognition identity, self-occupation stereotypes were significantly correlated with the other three dimensions of professional identity and personal control sense, *p*s < 0.01. Personal control sense was significantly correlated with professional identity and turnover intention, *ps* < 0.01. Besides, the four dimensions of professional identity all were significantly correlated with turnover intention, *ps* < 0.01. Such significant correlation results provided statistical support for the following mediation model testing (Mackinnon et al., 2000). Considering that three demographic variables (age, working age, and education) in Table 1 were significantly with self-occupation stereotypes or turnover intention, they were included in the SEM as controlled variables.

**TABLE 1:** Means, standard deviations, and correlations between variables.

Variables	1	2	3	4	5	6	7	8	9	10
1. Self-occupation stereotypes	1									
2. Personal control sense	−0.43**	1								
3. Professional cognition identity	–0.040	0.40**	1							
4. Professional emotion identity	0.44**	0.56**	0.41**	1						
5. Professional desire identity	0.34**	0.45**	0.39**	0.44**	1					
6. Professional desire identity	0.39**	0.46**	0.32**	0.56**	0.50**	1				
7. Turnover intention	0.39**	−0.58**	−0.41**	−0.63**	−0.53**	−0.64**	1			
8. Age	0.21**	0.12**	0.23**	0.14**	0.10**	0.22**	−0.22**	1		
9. Working age	0.22**	0.04	0.19**	0.02	0.10**	0.13**	−0.11**	0.64**	1	
10. Education	0.15**	–0.03	0.05	–0.05	−0.08**	−0.09**	0.03	0.03	0.19**	1
*M*	3.00	4.48	4.56	3.15	3.92	3.00	1.67	27.98	1.59	2.06
*SD*	1.12	1.04	0.68	0.83	0.89	1.01	0.92	7.61	1.00	0.53

N = 688. **p < 0.01. For the education of participants, 1 = High middle school or below, 2 = Junior college, 3 = undergraduate college, and 4 = Postgraduate.

### The Model Testing

The independent and serial mediating roles of personal control sense and professional identity between occupation stereotypes and turnover intention were analyzed in AMOS 23.0. Prior to the analysis, the normality of data was assessed in AMOS 23.0. According to the proposition by previous researchers (Kline, 1998), it is acceptable for the normality assessment if the skewness coefficient is less than 3 and the kurtosis coefficient is less than 8. Applying this criterion, the current variables showed an acceptable normality with the absolute values of the skewness coefficients ranging from 0.11 to 1.68 and the absolute values of the kurtosis coefficients ranging from 0.06 to 2.76. So, using the maximum likelihood estimation, we employed the mediation model based on 1,000 bootstrapped samples and 95% confidence intervals. The model was considered to be good when *CFI* and *TLI* ≥ 0.9, *RMSEA* ≤ 0.08, and *SRMR* ≤ 0.08 (Hu and Bentler, 1999).

After controlling for demographic variables, the results showed an acceptable model fit: *CFI* = 0.96, *TLI* = 0.91, *RMSEA* = 0.08, and *SRMR* = 0.07. As shown in Figure 1, self-occupation stereotypes were indirectly related to turnover intention via personal control sense and professional identity. When considering the indirect effects, the direct effect between self-occupation stereotypes and turnover intention was not significant. All indirect and indirect effects were shown in Table 2. As we have expected, self-occupation stereotypes were negatively related to personal control sense (β = −0.45, *p* < 0.001), and personal control sense was negatively related to turnover intention (β = −0.1, *p* < 0.05). The independent mediation of personal control sense showed a small but significant effect size (the effect size = 0.05, *p* < 0.05), thus supporting our hypothesis 2. In addition, self-occupation stereotypes also were significantly and negatively correlated with professional identity (β = −0.29, *p* < 0.05), and professional identity displayed a significantly negative correlation with turnover intention (β = −0.78, *p* < 0.01). Supporting our hypothesis 3, professional identity also mediated the relationship between self-occupation stereotypes and turnover intention with an effect size of 0.23 (*p* < 0.01). More importantly, self-occupation stereotypes were negatively correlated with turnover intention via the chain-mediating role of personal control sense and professional identity (the effect size = 0.18, *p* < 0.01), thus providing supports for our hypothesis 4. Overall, in general, self-occupation stereotypes held by private kindergarten teachers was positively related to their turnover intention. There were three indirect mediation paths underlying the relationship between them: (1) self-occupation stereotypes—personal control sense—turnover intention; (2) self-occupation stereotypes—professional identity—turnover intention; (3) and self-occupation stereotypes—personal control sense—professional identity—turnover intention.

**TABLE 2:** Direct and indirect effects between self-occupation stereotypes and turnover intention in the multiple model.

Effect	β	*SE*	*p*	95%CI
**Direct effect**				
Self-occupation stereotypes—turnover intention	–0.03	0.04	0.37	[−0.11, 0.05]
**Indirect effects**				
Self-occupation stereotypes—personal control sense—turnover intention	0.05*	0.02	0.02	[0.01, 0.08]
Self-occupation stereotypes—professional identity—turnover intention	0.23**	0.03	0.002	[0.17, 0.30]
Self-occupation stereotypes—personal control sense—professional identity—turnover intention	0.18**	0.02	0.002	[0.14, 0.22]

*p < 0.05, **p < 0.01.

## Discussion

With 688 private kindergarten teachers as participants, the current research examined the relationship between the self-occupation stereotypes of private kindergarten teachers and the underlying mechanism in China-mainland. Consistent with previous research (Yang et al., unpublished, see text footnote 1), our results demonstrated that those teachers with negative self-occupation stereotypes were more likely to have the intention of turnover in the future. More important, besides the independent mediating role of personal control sense revealed by previous research (Yang et al., unpublished, see text footnote 1), our research further demonstrated the independent mediating role of professional identity and the chain-mediating role of personal control sense and professional identity. The current research carried theoretical and practical implications.

### Theoretical Implications

Going beyond previous research (Yang et al., unpublished, see text footnote 1), the present research provided a more comprehensive insight about the mechanisms behind the correlation of self-occupation stereotypes and the turnover intention of private kindergarten teachers. Besides the independent mediating role of personal control sense on the individual level, there were the independent mediating role of professional identity on the organization level and the chain-mediating role of both variables between occupation stereotypes and turnover intention. Indeed, in the present research, the independent mediating effect of personal control sense actually was relatively small, and this may be because the effect was more replaced by the chain-mediating effect of personal control sense and professional identity (Zhou et al., 2020). That is to say, lower personal control sense may be more likely to be indirectly related to turnover intention via professional identity than directly related to turnover intention. Past literature has found that private kindergarten teachers displayed a lower professional identity in comparison with public kindergarten teachers in China-Mainland, and there was a significant correlation between the lower professional identity and the turnover intention of private kindergarten teachers (Tan, 2009; Lin, 2019). The present research made a significant contribution to such previous literature by firstly revealing that the perceived unstable work environment and the lack of personal control sense may be inducing factors that reduce the professional identity level of private kindergarten teachers in China-Mainland. According to the propositions of the Job-Demands-Resources Model, the perceived unstable work environment can be regarded as a special kind of pressure and turnover in essence is a negative strategy responding to the pressure (Bakker and Demerouti, 2007; Pienaara and Witte, 2016). Under this vein, increasing social supports from family/organization may be an effective way to alleviate the possible negative downstream effects of occupation stereotypes, because prior research has demonstrated that social supports can help employees cope with job stress effectively (Lambert et al., 2016).

Another thing deserves our further discussion is why Chinese people prefer to work as a public kindergarten rather than a private kindergarten teacher. It cannot be denied that there are indeed some differences between public and private kindergarten teachers (Pan et al., 2018). In China-Mainland, working in public kindergartens generally means more relaxed environment, lower working intensity, and more salaries in comparison to working in private kindergartens (Lin, 2019). Despite this, however, we cannot ignore the effects of cultural and cognitive factors in contribution to Chinese people’s preference for being a public kindergarten teacher. In specific, Confucian culture, which has a significant influence on the spiritual pursuit of Chinese people, has put forward that “settling down and starting one’s career” is the most important thing in our life (Xu, 2018). According to the doctrines of Confucianism, an ideal job should be a job working for the government, also called working “inside the system.” Maybe for this reason, besides the education industry mentioned in the present research, people’s preference for the job “inside the system (getting salaries from government)” widely exists in various of industries. We guess, the above realistic and cultural factors may both contribute to Chinese people’s fanatical pursuit for a public kindergarten teacher.

### Practical Implications

In practice, the current research provides a meaningful reference for how we alleviate the continuous hemorrhage of private kindergarten teachers. According to our results, some private kindergarten teachers may have held negative self-occupation stereotypes when entering the kindergarten. Past research suggests that although stereotype effects are robust, they actually can be alleviated and even removed by specific counter-stereotype training (Dasgupta and Asgari, 2004; Prati et al., 2015). So, to reduce self-occupation stereotypes of private kindergarten teachers, we can attempt to infiltrate some counter-stereotype examples into the career planning courses for those undergraduates majoring in preschool education, and the effect of the counter-stereotype training will be evaluated according to the preregistered schedule. In addition, our results suggested that the lower professional identity induced by occupation stereotypes was an important reason of the emergence of turnover intention for private kindergarten teachers. As we have mentioned above, improving the professional identity level of private kindergarten teachers by increasing social support toward teachers may be a feasible approach to alleviate the separation tendency of teachers. Indeed, several recent research has suggested that social support could improve the professional identity level of teachers, and alleviate their turnover tendency (Tang et al., 2015; Chen et al., 2020).

### Limitations and Future Work

The present research carried some limitations. Firstly, for the purpose of uncovering possible inducing factors behind the high turnover rate of private kindergarten teachers, our research mainly focused on the negative contents of occupation stereotypes about private kindergarten teachers in comparison with public kindergarten teachers. However, past research indicates that stereotypes about a certain group often contain mixed positive and negative contents (Fiske et al., 2002). So, future research should conduct a more comprehensive investigation on the contents of occupation stereotypes about private kindergarten teachers. Secondly, due to lacking available scale for assessing occupation stereotypes, we developed the self-designed scale to assess the self-occupation stereotypes of private kindergarten teachers. Moreover, the results based on the single questionnaire survey in essence only revealed the correlations among variables rather than the causality, and there is so far no directly causal evidence between occupation stereotypes and turnover intention. So, to be precise, the relationship between self-occupation stereotypes and turnover intention in our research should be indicative rather than conclusive, and more efforts should be made to further confirm the relationship between the occupation stereotypes of private kindergarten teachers and their turnover intention. Finally, there still are some controversies about to what extent stereotypes are accurate (Ryan, 2003). According to the “kernel of truth” of stereotypes (Penton–Voak et al., 2006), although occupation stereotypes of kindergarten teachers may exaggerate the differences between private and public kindergarten teachers, it is undoubtedly that private kindergarten teachers generally do more work and get less salaries compared to public kindergarten teachers in China-Mainland (Shi, 2020). Given that, we can attempt to distinguish the effect of the “actual differences” from the “perceived differences” in the future, which will be helpful for us to more accurately estimate to what extent the self-occupation stereotypes of private kindergarten teachers contribute to their turnover.

## Conclusion

With 692 private kindergarten teachers as participants, the current research demonstrated the significantly positive relationship between the self-occupation stereotypes of private kindergarten teachers and turnover intention in China-Mainland. For such private kindergarten teachers, negative self-occupation stereotype may induce lower personal control sense and lower professional identity level, and thus further leaded to the generation of turnover intention. The current research firstly clarifies the acting paths between the self-occupation stereotypes of private kindergarten teachers and turnover intention on both the individual and the organization levels. In practice, the research provides a novel perspective for policy makers to alleviate the turnover tendency of private kindergarten teachers. In the future, researchers should conduct laboratory experiments to provide more compelling evidence for the relationship between occupation stereotypes and turnover intention. And if possible, future research can also attempt to estimate to what extent occupation stereotypes represent the actual differences between private and public kindergarten teachers.

## Data Availability Statement

The raw data supporting the conclusions of this article will be made available by the authors, without undue reservation.

## Ethics Statement

All procedures performed in studies involving human participants were in accordance with the ethical standards of the Taishan University and with the 1964 Helsinki Declaration and its later amendments or comparable ethical standards. The patients/participants provided their written informed consent to participate in this study.

## Author Contributions

ML provided the idea that occupation stereotypes may cause the turnover intention of private kindergarten teachers, and then designed the research. YH collected the data included in the manuscript. FY conducted data analyses, wrote the manuscript, and made the subsequent revisions. All authors contributed to the article and approved the submitted version.

## Conflict of Interest

The authors declare that the research was conducted in the absence of any commercial or financial relationships that could be construed as a potential conflict of interest.

## Publisher’s Note

All claims expressed in this article are solely those of the authors and do not necessarily represent those of their affiliated organizations, or those of the publisher, the editors and the reviewers. Any product that may be evaluated in this article, or claim that may be made by its manufacturer, is not guaranteed or endorsed by the publisher.

## Footnotes

1 Yang, F., Han, Y., and Li, M. (2021). *The Effects of Occupation Stereotypes on the Turnover Intention of Private Kindergarten Teachers in China-Mainland: A Moderated Mediation Model (Unpublished Manuscript)*.
